# The impact of replacing wheat flour with cellular legume powder on starch bioaccessibility, glycaemic response and bread roll quality: A double-blind randomised controlled trial in healthy participants

**DOI:** 10.1016/j.foodhyd.2020.106565

**Published:** 2021-05

**Authors:** Balazs H. Bajka, Ana M. Pinto, Jennifer Ahn-Jarvis, Peter Ryden, Natalia Perez-Moral, Alice van der Schoot, Costanza Stocchi, Catherine Bland, Sarah E. Berry, Peter R. Ellis, Cathrina H. Edwards

**Affiliations:** a Biopolymers Group, Department of Biochemistry, Department of Nutritional Sciences, Faculty of Life Sciences and Medicine, King's College London, Franklin-Wilkins Building, 150 Stamford Street, London, SE1 9NH, UK; b Food Innovation and Health, Quadram Institute Bioscience, Norwich Research Park, NR4 7UQ, Norwich, UK; c Diet and Cardiometabolic Group, Department of Nutritional Sciences, Faculty of Life Sciences and Medicine, King's College London, Franklin-Wilkins Building, 150 Stamford Street, London, SE1 9NH, UK

**Keywords:** PulseON®, Legume, Plant cell, Type 1 resistant starch, Glycaemic response, Bread, Continuous glucose monitoring

## Abstract

The global rise in obesity and type 2 diabetes has generated significant interest in regulating the glycaemic impact of staple foods. Wheat breads (white or wholemeal) are popular staples, but have a high-glycaemic index, due to the highly digestible wheat starch. Reducing the glycaemic potency of white bread is challenging because the bread-making conditions are mostly conducive to starch gelatinisation. Cellular legume powders are a new source of type 1 resistant starch, where the starch is encapsulated by dietary fibre in the form of intact plant cell walls. The starch in these cell powders is less susceptible to gelatinisation and digestion than starch in conventional legume flours. However, legume cell resilience to baking conditions and the effects of this ingredient on glycaemic responses and product quality are unknown. Here we show that the integrity of cell wall fibre in chickpea powder was preserved on baking and this led to a ~40% reduction in *in vivo* glycaemic responses (iAUC_120_) to white bread rolls (~50 g available carbohydrate and 12 g wheat protein per serving) when 30% or 60% (w/w) of the wheat flour was replaced with intact cell powder. Significant reductions in glycaemic responses were achieved without adverse effects on bread texture, appearance or palatability. Starch digestibility analysis and microscopy confirmed the importance of cell integrity in attenuating glycaemic responses. Alternative processing methods that preserve cell integrity are a new, promising way to provide healthier low glycaemic staple foods; we anticipate that this will improve dietary options for diabetes care.

## Highlights

• Intact cell powder (ICP): a new legume ingredient increased type 1 resistant starch in bread rolls.
• ICP lowered starch bioaccessibility and glycaemic response of wheat bread rolls.
• Legume cell integrity was retained after baking and digestion.
• Wheat breads incorporating ICP had acceptable quality attributes.

## Graphical abstract

**Figure:**
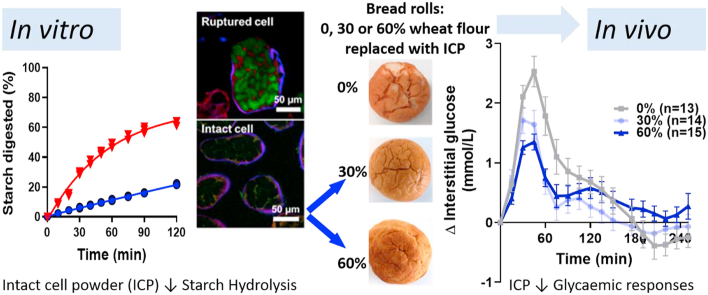

## 1 Introduction

The prevalence of obesity, type 2 diabetes and other cardiometabolic diseases is increasing rapidly and represents a major socio-economic burden worldwide (Hex, Bartlett, Wright, Taylor, & Varley, 2012). There is now convincing evidence that high glycaemic foods are contributing to increased incidence of type 2 diabetes and new strategies are required for cost-effective development of healthier carbohydrate foods (Livesey et al., 2019). Low glycaemic index (GI) foods could support the prevention and management of type 2 diabetes and benefit cardiometabolic health (Blaak et al., 2012; Jenkins et al., 2002; Livesey et al., 2019; Ludwig, 2002; Unwin, Haslam, & Livesey, 2016). The quantity and quality of dietary carbohydrates (e.g., starch and dietary fibre) has a major impact on postprandial glycaemia and insulinaemia (Ludwig, Hu, Tappy, & Brand-Miller, 2018; Russell et al., 2016). Carbohydrate staple foods, including many types of wheat bread, rice and potatoes, tend to have a high GI, whereas pulses (e.g., chickpeas, beans and lentils) can have a low GI and improve glycaemic control in healthy and diabetic individuals (Sievenpiper et al., 2009), but remain underutilised (Roberts et al., 2018). Improving the provision of highly palatable and convenient low-glycaemic staple foods is urgently needed for successful diet-based prevention/management of type 2 diabetes world-wide (Augustin et al., 2015).

The low glycaemic response to pulses occurs when the starch-containing cotyledon cells remain structurally intact after hydrothermal processing, such that the cell walls (comprising non-starch polysaccharides or ‘dietary fibre’) protect intracellular starch from digestive enzymes (Bhattarai, Dhital, Wu, Chen, & Gidley, 2017; Dhital, Bhattarai, Gorham, & Gidley, 2016; Golay et al., 1986; Grundy et al., 2016; Jarvis, Briggs, & Knox, 2003; Pallares Pallares et al., 2018; Rovalino-Córdova, Fogliano, & Capuano, 2019; Würsch, Del Vedovo, & Koellreutter, 1986). The important role of cell integrity during digestion has been previously demonstrated *in vivo* (Edwards et al., 2015; Ellis et al., 2004; Noah et al., 1998; Petropoulou et al., 2020). In the most recent study, intact pea plant cells aspirated from the human upper-gastrointestinal tract had limited permeability to amylase, resulting in reduced glucose availability from starch in the small intestine (Petropoulou et al., 2020). Conventional dry-milling and high temperature processing causes cell rupture, giving rise to products with highly digestible starch (Edwards, Maillot, Parker, & Warren, 2018; Li et al., 2020; Pallares Pallares, Loosveldt, Karimi, Hendrickx, & Grauwet, 2019; Verkempinck, Pallares Pallares, Hendrickx, & Grauwet, 2020) which evokes a high glycaemic response (Golay et al., 1986; Landa-Habana, Piña-Hernández, Agama-Acevedo, Tovar & Bello-Pérez, 2004; Tovar, Granfeldt, & Björck, 1992). Thus, retaining low glycaemic and resistant starch during incorporation of pulses into highly processed food products is a challenge.

A new proprietary process has been developed that enables the preservation of plant cell wall integrity during transformation of whole pulses into leguminous powders (Butterworth et al., 2019). The novel cellular powders obtained through this process contain high levels of type 1 (‘encapsulated’) resistant starch, where the intact cell walls (dietary fibre) act as a physical barrier to amylase and limit the rate and extent of starch digestion. The intact dietary fibre structure of plant cell walls also prevents complete hydration and gelatinisation of the encapsulated starch during hydrothermal processing, and therefore provides a new unexplored opportunity to preserve high levels of type 1 resistant starch in highly processed food products (Delamare et al., 2020; Edwards et al., 2020).

Wheat bread is a widely consumed starch-staple food and its transformation into a healthier product is likely to positively influence public health (Roberts et al., 2018). Typically, white bread is a high glycaemic index food (Foster-Powell, Holt, & Brand-Miller, 2002), because the temperature and moisture of the bread core (‘crumb’) during baking is conducive to wheat starch gelatinisation, which presents a challenge for incorporation of resistant starches (Fardet, Leenhardt, Lioger, Scalbert, & Rémésy, 2006; Roman & Martinez, 2019). We hypothesise that novel legume cell powders, unlike conventional flour, should retain cellular integrity and thus starch resistance under bread-making conditions, leading to a beneficial attenuation of glycaemic responses to wheat bread.

The aim of the study was to test the resilience of type 1 resistant starch in legume intact cell powders to the bread-making process, and subsequently investigate the glycaemic and product quality effects of bulk incorporation of legume intact cell powder as a wheat flour replacement in white wheat bread. Human studies were performed in combination with *in vitro* studies to provide further insight of the mechanisms of action of dietary fibre, as structurally intact cell walls, in producing type 1 resistant starch and attenuating amylolysis. This investigation has important implications for gastrointestinal and cardiometabolic health especially in relation to glycaemic control. Successful attenuation of glycaemic responses to bread without compromise on quality and cost, represents a significant opportunity for the food industry to create a new generation of food products to support dietary management of type 1 and 2 diabetes mellitus.

## 2 Materials and methods

### 2.1 Bread roll ingredients

In these experiments we evaluated the performance of chickpea PulseON®, an intact cell powder (ICP), as an ingredient in wheat bread rolls. The ICP was prepared by New Food Innovation Ltd. (UK) from whole chickpeas (*Cicer arietinum* L., ~7 mm size, Kabuli-type, Argentine variety, supplied by AGT Poortman Ltd., UK) according to a proprietary process (PCT/GB2019/ 050284) involving hydrothermal processing, wet-sieving and drying (Butterworth et al., 2019). The ICP used in this study contained 19.5% protein, 6.5% fat, 48.0% starch, 2.0% total sugars, 20% dietary fibre, and 3.6% moisture, and 1461.8 kJ per 100 g, analysed according to methods described in Section 2.3. Further physico-chemical characteristics of this ingredient (PulseON®) have been described in detail elsewhere (Delamare et al., 2020; Edwards et al., 2020).

The chickpea ruptured cell powder (RCP) was prepared by blending ICP in a Thermomix® TM5 (Vorwerk UK Ltd., Sunninghill, UK) and passing it through a 75 μm aperture analytical sieve. The resulting cell powder (RCP) had a smaller particle size with a higher proportion of ruptured cells and was included for comparison in Study 1 to enable further mechanistic insights into the role of cellular integrity on starch digestibility and glycaemia. The particle size of this ingredient was confirmed through laser diffraction by a method that assumes particles are a spherical shape, as described previously (Edwards et al., 2020).

Other ingredients used to prepare the bread were: wheat flour (Taste the Difference Very Strong Canadian Bread Flour, Sainsbury's, London, UK); sucrose (white caster sugar, Sainsbury's, London, UK); NaCl (Saxa table salt, Premier Foods, St Albans, UK); vegetable fat (Trex Vegetable Baking Fat, Princes Group, Liverpool, UK) purchased from Sainsbury's supermarket, Norwich, UK; wheat gluten (Vital Wheat Gluten 75–80% protein, Bob's Red Mill, Milwaukie, US) and ascorbic acid (Dove's farm, Hungerford, UK), purchased from Amazon; and dry Baker's yeast (Ferminpan Red, Lallemand, Felixstowe, UK) provided by Lallemand. The wheat flour used in this study contained approximately 14.8% protein, 1.3% fat, 65.9% starch, 1.4% total sugars, 3% dietary fibre, and 13.6% moisture, and 1468 kJ per 100 g and was a major source of digestible starch and wheat protein (mainly as gluten) in the bread roll recipes. The vital wheat gluten contained approximately 76.7% protein, 1.7% fat, 10% starch, 3.3% dietary fibre and 8.3% moisture, and was a further source of wheat protein and starch, particularly in the ICP-enriched rolls.

### 2.2 Bread roll preparation

All bread rolls were prepared in a clinical test kitchen (NHS Clinical Research Facility, Norwich, UK). Three bread rolls, each containing different doses of chickpea ICP were produced; these were denoted 0% (control), 30% and 60% bread, as chickpea ICP was added to the recipe at 0, 30 or 60 (w/w) % replacement of wheat flour.

All ingredients were measured and combined using the proportions described in Table 1. To ensure an accurate glycaemic load for the human Study 2, the quantity of ingredients in the recipe was altered to ensure that each bread roll contained the same amount of total starch (50 g). More gluten was added to the ICP rolls to replace the functionality of protein from the wheat flour and to achieve a similar amount of wheat protein for all roll types (~12 g per roll).

**Table 1:** Recipes for bread rolls made with 0, 30 and 60% intact cell powders (ICP)^a^.

Ingredients (g)	0% ICP		30% ICP		60% ICP	
	Sponge	Dough	Sponge	Dough	Sponge	Dough
Water	347.6	233.5	470.5	438.2	365.7	1009.9
Wheat flour	540.4	396.0	688.6	0.0	350.8	0.0
Vital gluten	14.7	0.0	49.9	0.0	141.4	0.0
ICP	0.0	0.0	0.0	317.5	0.0	739.9
Caster sugar (sucrose)	0.0	16.3	0.0	20.8	0.0	27.7
Vegetable fat	0.0	29.4	0.0	37.5	0.0	49.9
Baker's dry yeast	7.35	7.35	9.35	9.35	12.5	2.5
Ascorbic acid	0.0	2.0	0.0	2.5	0.0	3.3
Table salt (NaCl)	6.6	6.5	8.3	8.3	11.1	11.1

a Weight of each ingredient (g) added at the sponge- and dough-stage for preparation of 0% (Control) and 30% and 60% chickpea ICP bread rolls.

One 12-roll batch of each bread type (0, 30, and 60% chickpea ICP) was produced on each of two consecutive days using a sponge-dough process. Sponge ingredients (flour, gluten, yeast, and water) were mixed using a pre-weighed 6.9L heavy duty planetary mixer (model 5KSM7591XBSM, Kitchen Aid, Antwerp, BE) with a paddle attachment. The sponge was developed for 8 min, then transferred into resealable plastic bags and stored under ambient conditions (22.0 ± 3.0 °C). After 2 h, the remaining dry ingredients and water were combined with sponge mixtures in the planetary mixer (using a 6.9 L bowl and dough hook attachment) and allowed to develop for 8 min. The resulting dough was portioned into bread rolls and immediately proved in a pre-heated high-humidity (100% RH) combination oven (Rational Self Cooking Centre, Luton, UK) at 38 °C for 30 min. After proving the rolls were lightly spray misted with water and baked by convection at 185 °C for 15 min to achieve an internal core temperature of 95 ± 2 °C. Weight (moisture loss) after baking was recorded for each bread roll. Finished rolls were cooled for 2 h and stored frozen at −20 °C in resealable, opaque polyethylene bags (3 mm thickness). A third batch of rolls were produced under identical conditions at a later date to provide a third replicate for bread quality assessment (physical and sensory attributes).

### 2.3 Nutrient composition and microbiological safety

Proximate nutritional analysis of ICP and breads baked for the human dietary intervention study were performed by UKAS accredited testing at ALS Laboratories Ltd., Chatteris, Cambridgeshire, UK. Specifically, protein was quantified by Dumas using a Nitrogen conversion factor of 6.25, total fat by NMR, total dietary fibre by AOAC method no. 985.29 (‘Prosky method’), total sugars by ion exchange chromatography, available carbohydrates by ‘difference’ calculation, sodium by ICP-OES and energy was calculated using standard energy conversion factors (EC 2008/100 and 90/496). The dietary fibre measurement includes high molecular weight soluble and insoluble dietary fibres (e.g., pectin, arabinogalacatans, cellulose), but does not include some lower molecular weight soluble oligosaccharides (e.g., raffinose, stachyose), and resistant starch is believed to be only partially measured (McCleary et al., 2019).

Total and resistant starch content of ICP, RCP and bread products were determined in house using the reagents from ‘Total starch kit’ KTSTA-100A and ‘Resistant starch (rapid) kit’ K-RAPRS, Megazyme International, Wicklow, Ireland. Total starch analysis was performed using the smaller-scale DMSO version of the method, as described previously (Edwards et al., 2015). The sum of the measured total starch and sugar content is referred to in this manuscript as ‘potentially available carbohydrate’ and was used for calculation and analytical purposes rather than the less accurate ‘by difference’ method. We consider that these carbohydrates have the biochemical potential to be digested into simple sugars by enzymes in the human upper-gastrointestinal tract, but some of the starch may exhibit slow and/or resistant digestion behaviour due to extrinsic (cell wall) factors, and are therefore best described as ‘potentially available’.

Microbiological safety testing of bread rolls was performed by ALS Laboratories Ltd. prior to the human intervention study and included total viable counts (after 2 days) of *Enterobacteriaceae*, *Escherichia coli*, *Clostridium perfringens, Staphylococci* (coagulase positive), *Bacillus cereus*, *Salmonella* spp.*,* yeasts and moulds. All microbiological counts were within levels deemed safe for human consumption; no *Salmonella* spp. were detected. The chickpea ICP was also tested for trypsin inhibitors (AACC international method 22-40-01 (AACC, 2000)) and lectins by haemagglutination test (Rodhouse, Haugh, Roberts, & Gilbert, 1990) by Campden BRI, Chipping Campden, UK. Lectin and trypsin inhibitor levels in ICP were below the lower limit of quantification (<120 haemagglutination units/g sample and <3.1 trypsin inhibitor units/mg sample).

### 2.4 Bread roll quality assessment

For each bread type (i.e. dose of ICP), a representative subset of three bread rolls from each of the three separate bake days were subjected to quality analyses. Bread rolls were defrosted in their packaging at room temperature (22 ± 2 °C) for exactly 12 h prior to quality analyses, which included assessment of specific volume, crumb and crust colour, and texture. These tests were done in the same order for each treatment and completed within 2 h to control for quality variation as a result of starch retrogradation and/or drying.

Bulk density (g/cm^3^) was determined using AACC method 10-05 (volume based on rapeseed displacement) and the roll mass (g) (AACC, 2000). A metal block (5 x 5 × 5 cm) was used as the standard. Bread roll mass was determined immediately prior to rapeseed displacement measures.

Instrumental measurement of crumb and crust colour was achieved using a bed scanner (Perfection V850 Pro, Seiko Epson Corp., Suwa, JP). For each bread roll type, L*, a* and b* values were obtained using Adobe Photoshop CS6 extended software (San Jose, US) from three rolls with eight readings from each roll at different locations on the crumb and crust. Hue angle and chroma were calculated (Maskan, 2001).

Bread roll crumb texture was characterised with a texture profile analysis two-bite test on a Stable Micro Systems texture analyser (TAXT2) equipped with a 5 kg load cell and using a modification of the AACC method 74-09 (bread firmness by universal testing) (AACC, 2000). Samples were compressed in a dual cycle using a 50 mm diameter cylinder probe (P50) with a crosshead speed of 10 mm min^−1^. This was applied to 25 x 25 × 25 mm samples to mimic mastication; crumb ‘hardness’ corresponded to the maximum force required to achieve 40% compression. Five replicate measures were performed on each bread sample. Stable Micro Systems Exponent (version 6.0, Stable Micro Systems, Godalming, UK) software was used to calculate ‘springiness’ and ‘chewiness’ as defined by Szczesniak (Szczesniak, 2002) and applied to bread (Scanlon, Sapirstein, & Fahloul, 2000).

### 2.5 Microscopy

For light microscopy, images were captured using an Olympus BX60 Microscope equipped with a Jenoptik 232 ProgRes camera. Samples were taken before and after digestion and stored in 10% formalin solution until required. A small amount of Lugol's iodine (I_2_/KI) solution was added to the samples to stain the starch before placing the suspension in a slide with a glass cover and examined.

For confocal imaging of the chickpea powders, RCP and ICP were suspended in PBS containing 10 μg/mL calcofluor-white, fluorescein isothiocyanate (FITC) and fast green FCF (Sigma-Aldrich Co, Poole, UK) to stain cell walls, starch and protein, respectively. Following incubation for 30 min at room temperature on a rotating mixer, samples were centrifuged and washed twice in PBS, mounted on slides and viewed under a Leica SP2 confocal microscope (Leica Microsystems, Mannheim, Germany). Samples were imaged using laser excitations of 405 nm, 488 nm and 633 nm and emissions of 410–480 nm, 500–580 nm and 640–750 nm for calcofluor, FITC and fast green, respectively. Image stacks of chickpea cells at 10x magnification and a representative chickpea cell at 63x magnification, and average z-projections and image analysis were conducted using Fiji image analysis software (http://fiji.sc/). Images on the diffusion of FITC-amylase were obtained in a Zeiss LSM880 confocal laser scanning microscope and processed using ZEN Blue software. FITC-labelled amylase was prepared by mixing pancreatic α-amylase dissolved in 0.1M sodium carbonate buffer pH 9 with FITC in the same buffer at a molar ratio 1:100. After stirring in the dark for 3 h at room temperature, the labelled enzyme was purified in a PD-10 column equilibrated with PBS at pH 7.4. Samples of cooked chickpea cells were stained with calcofluor white and fast green FCF as described before and incubated with FITC-amylase at 37 °C for 2 h.

### 2.6 *In vitro* starch digestibility (amylolysis)

Cell powders and representative bread rolls from each treatment and batch were subjected to starch digestibility analysis (amylolysis) using published methods (Edwards, Cochetel, Setterfield, Perez-Moral, & Warren, 2019; Edwards et al., 2020). Bread roll samples (1–2 g) were obtained from the same subset of bread rolls used in the quality assessments; they were blended in a coffee grinder (KRUPS F20342, 3 × 5 s, with 5 s rests between) and sieved to obtain crumbs (1–2 mm diameter), which were alternately weighed out into dry-tared aluminium pans for moisture analysis (triplicates of ~350 mg) or 15 mL Corning centrifuge tubes for quadruplicate digestibility assays. The weight of each bread roll sample used for digestibility analyses was chosen with the aim of obtaining a constant amylase: starch ratio of 0.89 amylase U/mL:5 mg starch/mL in the final digestion mixture. Moisture content was then determined from weight loss following oven-drying for 16 h at 103 °C (Binder Model ED-56). Starch digestibility was determined by the ‘pahbah’ (*p*-hydroxybenzoic acid hydrazide) assay for reducing sugar analysis of samples withdrawn after 0, 10, 20, 30, 40, 50, 60, 75, 90 and 120 min of incubation with porcine pancreatic α-amylase, as described previously (Edwards et al., 2019). Amylolysis assays of the chickpea powders were performed in triplicate, and assays of bread rolls performed in quadruplicate. The curves showing starch amylolysis digestion were baseline corrected by subtracting the value at *t* = 0 min from subsequent measures done within the same sample tube. Values for the first-order rate constant, *k* and endpoint*, C*_∞_, values were obtained as described elsewhere (Edwards, Warren, Milligan, Butterworth, & Ellis, 2014). As the *k* and *C*_*∞*_ parameters are covariant, contour plots (wherein the black contour shows residual sum of squares greater than the minimum by a factor of 1.5) were obtained in R Core Team (R Foundation, 2013, Vienna, Austria) and used to illustrate the joint probability of distinct digestion behaviours.

### 2.7 Acute human dietary intervention studies

#### 2.7.1 Study designs

Two double-blind randomised controlled studies, with a three-arm cross over design, were undertaken at the metabolic research unit (MRU) of King's College London between June 2019 and January 2020. The trials investigated the effects of chickpea ICP (consisting predominantly of intact cells) on glycaemia. These studies were registered at clinicaltrials.gov as NCT03994276 Study 1 tested the postprandial glycaemic response to drinks containing 50 g of carbohydrate (mainly starch) in the form of ICP and RCP, and a reference drink containing 50 g carbohydrate as glucose. Study 2 tested the postprandial glycaemic response to bread rolls incorporating different quantities of ICP into the recipe (i.e., 0, 30 or 60% w/w of wheat flour replaced with ICP) and formulated to contain the same amount of potentially available carbohydrate per serving.

The studies were conducted in accordance with the Declaration of Helsinki and approved by the relevant research ethics committee in the UK (#HR-18/19–8431, BDM Research Ethics Subcommittee at King's College London). All participants' data were stored in accordance with the General Data Protection Regulation 2018 and biological samples were handled, stored, transported and disposed of in accordance with the Human Tissue Act (2004). Participants were reimbursed for their time and travel expenses upon completion of each study.

#### 2.7.2 Study participants

Healthy participants aged 18–45 years were recruited using advertisements around King's College London, including circular e-mails and posters. Exclusion criteria included: body mass index (BMI) < 18 or >35 kg/m^2^, blood pressure ≥ 160/100 mmHg, fasted glucose > 6.0 mmol/L, plasma cholesterol ≥ 7.8 mmol/L, plasma triacylglycerol ≥ 5.0 mmol/L, medications that may interfere with the study (e.g. antidiabetic or lipid-lowering drugs), allergy or sensitivity to wheat, alcohol intake >28 units/week and active or recent cessation of smoking (<6 months). Participants were healthy with no history of cardiovascular disease, diabetes or gastrointestinal disorders, as determined by a pre-screening health questionnaire. Body mass index (BMI), blood pressure, liver function, blood cell count, fasting plasma glucose and lipid concentrations were confirmed to be within normal limits during a clinical screening visit that took place before confirming enrolment.

The characteristics of participants that were enrolled onto each study are reported in Table S.1*.* In study 1, all 20 enrolled participants were initially fitted with devices for monitoring of glucose concentrations in interstitial fluid, in addition to capillary blood collection during the postprandial period. For study 2, 21 participants were enrolled onto the study, of which 16 were fitted with continuous glucose monitors. In both studies, issues with these monitors (failure of sensors or devices, data recording and/or download) and drop-outs meant that the final data analyses were performed with data from fewer than the enrolled number of participants. The CONSORT diagrams in Fig. S.1 and S.2 provide further details of the flow of participants through the study. The number of participants (n) whose data were included in the analyses are reported together with mean values, graphs and statistical comparisons*.*

#### 2.7.3 Dietary interventions

Study 1 assessed the acute 2 h postprandial glycaemic response to three test drinks (i.e. glucose, ICP or RCP) all providing 58 g potentially available carbohydrate (50 g from the ICP or RCP plus 8 g carbohydrate in the flavouring to aid palatability). Potentially available carbohydrate was provided as 58 g glucose in the form of dextrose powder from Thornton & Ross, Linthwaite, UK); or as starch (50 g) from chickpea powder (100 g ICP or RCP), plus 11 g Nesquick chocolate powder with no added sugar (Nestlé, Verve, CH) for flavouring. The moisture contents of all powders were determined by oven-drying method and weights of powder per serving adjusted accordingly to achieve 58 g potentially available carbohydrate per serving. Complete dispersion of the powders was ensured by the use of a protein shaker bottle containing a blender ball. Additionally, participants were asked to shake the bottle periodically during consumption. All drinks were made up to 330 mL with bottled water (Tesco, Ashbeck, UK). Time to drink was recorded and participants were encouraged to drink at the same rate for all 3 test drinks.

Study 2 assessed the 4 h postprandial glycaemic response to three bread roll types in which 0, 30 or 60%, w/w, of the wheat flour in the recipe was replaced with chickpea ICP. Replacement of wheat flour with ICP meant that ~12 and 30 g of the total starch in the 0% bread roll was replaced by starch from ICP to make the 30% and 60% bread rolls, respectively. Bread rolls were prepared as described in Section 2.2. The roll mass and the amount of gluten added was adjusted for each bread type to compensate for the lower starch and gluten content of the ICP. This ensured that all bread roll types appeared similar and contained the same amount of starch and wheat protein per serving (= 1 roll). Each bread roll was served with 20 g of no-added sugar strawberry jam (Energy Reduced Strawberry Jam with Sweetener, Marillo Foods Ltd., West Yorkshire, UK) providing <0.4 g sugars (mainly fructose) and 12.8 g polyols (mainly from sorbitol), to aid palatability. As the different type of breads had different weights, due mostly to the moisture content, the total weight of drinking water served with each meal was adjusted in order to achieve a constant total weight of 420 g. Participants were instructed to consume the meal at their normal pace which was standardised based on their first visit. Each participant received a different bread roll treatment on each of three separate visits to the Metabolic Research Unit (MRU) in the Department of Nutritional Sciences, King's College London. The order in which they received the treatments was allocated randomly using Sealed Envelope™ (version 1, Sealed Envelope Ltd., London, UK). A coded system ensured that investigators assessing outcomes were blinded to avoid experimenter bias.

In both studies, participants were fitted with a continuous glucose monitor (CGM, Freestyle Libre, Abbott Laboratories Ltd, Elgin, US) capable of recording subcutaneous glucose concentrations every 15 min continuously for up to 2 weeks. The CGM was covered by a protective film and applied to the back of the upper arm at least 12 h before the first visit and remained in place throughout the duration of study 1. Two monitors per participant were used for study 2 as the total study duration exceeded the 14-day recommended period of use of the sensors. On the morning of each intervention, the participants arrived at the MRU after a 12 h fast and having consumed a standard meal that was provided to them (350–450 kcal and <12 g fat per serving, and <3 g dietary fibre per 100 g) the previous evening. Participants marked responses to palatability questions (enjoyment; difficulty to consume the given volume; taste; moisture; texture; aftertaste) using 100 mm Visual Analogue Scales (VAS) at baseline (t = −10), immediately after the test or control meal, and at 30, 60, 90, 120, 180 and 240 min. The glucose data from the CGM was downloaded with an associated reader using Freestyle Libre Pro software (version 1.0, Abbott Laboratories, Alameda, US) at the end of each visit. Time stamped glucose concentrations were recorded for the 120 min postprandial period, including a baseline measurement 15 min prior to consumption of the allocated test meal (t = 0). In study 1, additional capillary blood glucose measurements were obtained by finger-prick method using an Accu-Chek Performa-nano monitor with test strips (Roche Diabetes Care Ltd, Mannheim, Germany) and lancets (GlucoRx, Surrey, UK).

#### 2.7.4 Data analysis

Time points from the CGM recordings were anchored at t = 0, defined as the closest reading to the time of the participants first bite of the breakfast test meal, and selected every 15 min thereafter. Incremental postprandial glucose responses were obtained by subtracting the baseline glucose measurement(s) from subsequent glucose measurements within the same individual on each occasion. The incremental area under the interstitial glucose response curve (iAUC) up to a specified time point was calculated using only the area under the curve above the baseline (ignoring the area under baseline) according to the method referred to as ‘Method 3 – incremental AUC method’ in Brouns et al., (2005).

### 2.8 Statistical analysis

Statistical analyses and graphical representations were done using GraphPad Prism 8.4.1 software for Windows (GraphPad Software LLC, San Diego, US). *In vitro* data was analysed by repeated measures ANOVA, and *in vivo* data analysed by mixed model ANOVA, with time and treatment (e.g., bread roll type) as fixed effects and individual differences as random effects. The number of participants (*n*) whose data was included in each analysis is indicated in figure legends and throughout the text. Outliers were not excluded. Postprandial glucose response data are provided as ***Supplementary data,*** with mean, SD and *n* shown for each time point. A Greenhouse-Geisser correction was applied to correct for violations of sphericity. *Post hoc* analyses were performed when significant treatment x time effects were detected, and Tukey's correction for multiple comparisons applied (adjusted *p*-values reported). Statistically significant effects were accepted at the 95% confidence level. Mean values are reported with SD unless otherwise specified and violin plots indicate the distribution of the data.

## 3 Results & discussion

### 3.1 Cell integrity mechanism

Conventional dry-milled legume flours consist mostly of ruptured cells and exposed starch which becomes highly susceptible to digestion upon hydrothermal processing. The novel pre-processing treatment used to obtain ICP differs from conventional dry-milling in that it facilitates cell separation so that most of the starch in ICP remains enclosed by leguminous cell walls (Edwards et al., 2020).

The role of cell integrity in *in vitro* starch digestibility and *in vivo* glycaemia was determined by comparing the intact cell powder ‘ICP’ with a compositionally equivalent comminuted powder referred to as ‘ruptured’ cell powder, RCP. The RCP was obtained by grinding the ICP to pass a 75 μm sieve screen, and therefore contained a higher proportion of ruptured cells. The analysis of RCP was also of interest with regard to the commercial viability of the material; for instance, a smaller particle size may be desirable for some applications, and some cell rupture is likely to occur during large-scale industrial production of the powder.

**Fig. 1:**
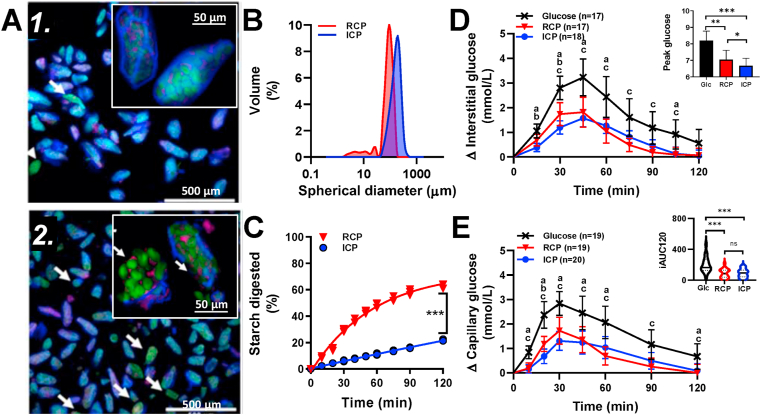
Cell powder characteristics and mechanism. Confocal images of intact (A1) and ruptured (A2) cell powders (‘ICP’ and ‘RCP’). Cell walls (blue), starch (green) and protein (red). Arrows highlight damaged cells. Particle size distributions (B); starch amylolysis (C) curves show highly significant differences between ICP and RCP; incremental glycaemic responses in interstitial fluid (D) and capillary blood (E) as means ± 95% CIs for number of participants (n) after drinks of glucose (‘glc’) ***X***, RCP ▼ and ICP ●, each containing 58 g of potentially available carbohydrate (predominantly as glucose in ‘glc’ or as starch in RCP and ICP). Bar chart (insert D) shows mean peak glucose concentration with 95% CIs. Violin plots (insert E) show distribution and median incremental area under the curves, ‘iAUC120’. Significant differences between drinks annotated: **a** – glc vs ICP; **b** – ICP vs RCP and **c**-glc vs RCP.

Imaging confirmed that the ICP consisted almost entirely of intact isolated cells (Fig. 1A.1), where the starch and protein were surrounded by undamaged cell walls. In the RCP, while many cells were intact, there was some cell damage (Fig. 1A.2, arrows) and release of partially swollen starch granules (~40 μm). Particle size analyses (Fig. 1B) confirmed that the majority of ICP particles were within the size range of single cells (Edwards et al., 2020), with some cell clusters. Particles in RCP were also mainly within the cellular size range (up to 89 %), but the small peak at 40 μm with a tail to 2 μm is consistent with cell rupture and starch granule release. We estimate that at least 11% of RCP particle volume was below the cellular range.

The susceptibility of starch to digestion by α-amylase is known to impact on glycaemia (Edwards et al., 2019) and was determined using an *in vitro* amylolysis assay. The RCP had significantly higher starch susceptibility to amylolysis than ICP (ingredient × time interaction *F*_9,36_ = 1226, *p* < 0.0001). After 120 min of amylolysis, 63.0 ± 1.7% of the carbohydrate (starch) had been digested in RCP, compared with 21.7 ± 0.6% in the ICP. Thus, the grinding operation used to obtain RCP led to a three-fold increase in the amount of bioaccessible starch. Overall, the progress of starch amylolysis in both ICP and RCP (Fig. 1C) was slow compared to a gelatinised chickpea or wheat flour (Edwards et al., 2020) and is characteristic of low-medium glycaemic materials (Edwards et al., 2019).

Mean CGM response profiles to each drink type (ICP, RCP and glucose) are shown in Fig. 1D. There were significant effects of time (*F*_1.77, 30.1_ = 45.72, *p* < 0.001) and drink type (*F*_1.34,22.7_ = 19.50, *p* < 0.001) on glycaemic responses, and a significant drink × time interaction (*F*_3.23, 50.1_ = 4.233, *p* = 0.008) over 120 min (Fig. 1D).

Cell integrity had a significant effect on the iAUC_45_, which was 40% higher after RCP (49.3 ± 28.8 mmol/L/min, n = 17) than ICP (35.1 ± 18.4 mmol/L/min, n = 18), *F*_1.797, 28.74_ = 27.38, *adj. p* = 0.044). Peak glucose concentrations (reached at ~45 min, irrespective of drink type) were significantly higher (*adj. p* = 0.028) after RCP (6.8 ± 1.5 mmol/L, n = 17) compared with the ICP (6.2 ± 1.2 mmol/L, n = 18), *F*_1.743, 27.89_ = 26.63 (Fig. 1D insert). Values obtained for iAUC_60_ and iAUC_120_ were not significantly different between RCP and ICP drinks (*p* > 0.05). These data indicate that differences in cell integrity influence primarily the early postprandial glucose response, and are consistent with the slower rate of release of starch amylolysis products from ICP compared with RCP, observed *in vitro*.

The acute incremental areas under the postprandial curves (iAUC_120_) for ICP and RCP were significantly smaller (by 52.7% and 55.8%, respectively), compared with the glucose reference (*F*_1.25, 19.9_ = 20.20, *adj. p* ≤ 0.001). The attenuated glycaemic response to ICP and RCP are consistent with the *in vitro* data showing slower release of starch amylolysis products (glucose oligomers), and reduced glucose availability compared with the glucose reference drink.

Capillary blood glucose responses (Fig. 1E) followed similar trends to the CGM responses (Fig. 1D), with both ICP and RCP test drinks giving significantly lower glucose responses and smaller iAUC_120_ values (by 54.2% for ICP and 54.8% for RCP) than the glucose reference (meal x time effects, *F*_3.76, 65.6_ = 3.337, *p* = 0.0168). Peak capillary glucose concentration after RCP (7.0 ± 1.2 mmol/L, n = 19) and ICP (6.7 ± 1.0 mmol/L, n = 20) were significantly lower than after the glucose drink (8.2 ± 1.2 mmol/L, n = 19, F _1.52, 27.42_ = 19.03, *p* < 0.001). Although differences in peak glucose time were observed (30 min for capillary blood and 45 min for CGM), the correlations between capillary and CGM values at both 30 and 45 min were strong and highly significant (*r*^*2*^ = 0.728 and *r*^*2*^ = 0.806, *p* < 0.0001, respectively). The main limitation of the CGM technology was the 14% failure rate of the devices.

The differences between *in vivo* glycaemic effects of RCP and ICP were evident only in the first 45 min following test drink consumption, and were not as clear as seen *in vitro*. While the *in vitro* assay is highly sensitive to differences in starch susceptibility (Edwards et al., 2019), the limited starch availability from cell powders may have been insufficient to cause large enough perturbations in peripheral glucose concentrations to discriminate between RCP and ICP *in vivo*. A higher degree of cell rupture for RCP may give a larger and clearer *in vitro* starch digestibility and peripheral glucose response; however, this would affect test drink palatability and participant compliance. Nevertheless, the larger increase in mean glucose concentration following RCP compared with ICP within the early postprandial stage (first 30 or 45 min following ingestion), is likely the result of higher starch bioaccessibility (observed *in vitro*, Fig. 1C) from ruptured cells (Fig. 1A2). These data support the hypothesis that cell integrity is an important underpinning mechanism, and that preservation of cell integrity through downstream processing is necessary to limit starch bioaccessibility from these novel flours.

### 3.2 Effects on bread roll quality

To investigate further the potential for preserving cellular integrity in a baked bread product, we used the ICP to substitute 0 (‘control’), 30 or 60% (w/w) of the white wheat flour in a white wheat bread recipe, and determined the effects on microstructure and physical characteristics of the final baked product. These results are presented together with the palatability scores that were collected from study participants (Table S.1 for ‘Study 2 participant characteristics’) during the intervention visits in human study 2 (Fig S.2 – ‘Study 2 CONSORT diagram’).

Bread rolls of each type (Table 1) were baked to provide a target 50 g starch per single roll serving, and average baked roll masses were 115, 150 and 201 g for 0, 30 and 60% breads respectively (CV of 1.4, 1.8% and 0.7%, respectively). Proximate analyses (Table 2 and Table S.2) suggested that each bread roll type provided approximately 50 g starch (by difference) and 5 g sugar (mainly maltose) per serving, irrespective of the proportion of ICP used. Values obtained by direct analysis of total starch suggest that the achieved potentially available carbohydrate content was slightly lower than the target, but consistent across all rolls at 48 g per serving. Wheat flour/gluten provided 75% of the total starch in the 30% bread roll, and 41% of the starch in the 60% bread roll, with the remaining starch provided by the ICP.

**Table 2:** Nutrient composition of bread rolls (per served portion).

	0% ICP	30% ICP	60% ICP
Serving Size (g)	115.0 ± 2.7	150.0 ± 1.6	201.2 ± 1.3
Moisture (g) a	39.1	62.3	94.6
Energy (kJ) a	1301.8	1501.5	1823.1
Protein (g) a	12.88	17.7	27.16
*from wheat*^c^	*94%*	*65%*	*49%*
*from chickpea*^c^	*0%*	*29%*	*44%*
Total Fat (g) a	3.33	5.55	8.84
Total Starch (g)^b^	45.3	44.9	42.5
*from wheat*^c^	*100%*	*75%*	*41%*
*from chickpea*^c^	*0%*	*25%*	*59%*
*Digestible starch*^b^	*99.4%*	*96.4%*	*92.6%*
*Resistant starch*^b^	*0.6%*	*3.6%*	*7.4%*
Total Sugars (g)^a^	2.76	3.45	5.67
Potentially available carbohydrates (g) d	48.1	48.3	48.2
Dietary Fibre (g) a	2.64	6.15	10.65
Sodium (mg)^a^	418.6	543	751.7

a Proximate determinations by ALS Laboratories.

b Direct measurements obtained using Megazyme total and resistant starch kits.

c Estimated proportion of protein or starch originating from wheat (wheat flour, gluten) and chickpea (ICP) calculated from ingredient composition and amounts used in each recipe.

d ‘Potentially available carbohydrates’ is the sum of total starch and sugars. Data is shown for 0, 30 and 60% bread roll types, where the % refers to proportion of wheat flour replaced with ICP (w/w).

The Resistant Starch ‘rapid assay’ method (Megazyme) showed that 15.5% of the starch in the ICP was classified as ‘resistant’, and the proportion of ‘resistant starch’ (0.6, 3.6 and 7.4% of total starch) detected in control (0%), 30% and 60% breads, respectively, corresponded to the proportion of ICP used in each recipe. These data suggest that some forms of resistant starch in the ICP were preserved through bread-making, and the starch amylolysis experiments in the following section provide a more complete indication of bread starch digestion profiles.

A similar amount of wheat protein per serving (11.2–13.5 g) was achieved across all bread rolls by addition of vital gluten to replace gluten from the wheat flour. Because ICP has a lower starch content, but higher protein, fat, and dietary fibre content than wheat flour (Edwards et al., 2020), matching for carbohydrate load resulted in differences in total protein (12.9, 17.7 and 27.1 g/serving), total fat (3.3, 5.6 and 8.8 g/serving) and total dietary fibre (2.6, 6.2 and 10.7 g/serving) for the control (0%), 30 and 60% bread type, respectively. However, microstructural observations indicated that the additional chickpea protein (up to 12 g in the 60% roll) and fat from ICP is contained within the intracellular space, particularly during early stages of digestion, and therefore likely to be of limited influence on the glycaemic responses.

**Fig. 2:**
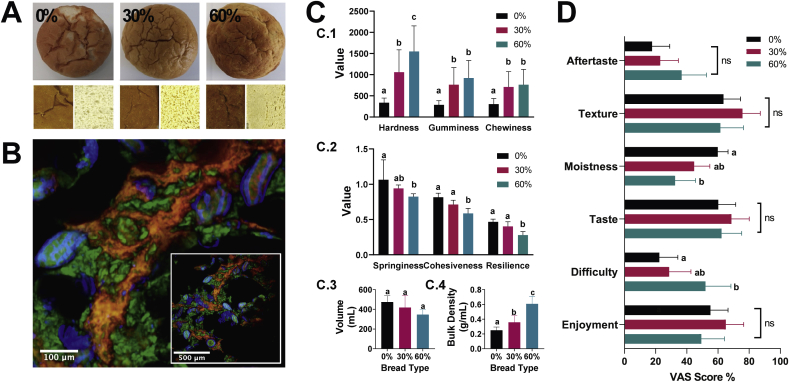
Physical characteristics and sensory qualities of control and test breads. Photographs (A) of bread roll crust and crumb for bread with 0, 30 or 60% wheat flour in the recipe replaced with intact cell powder (ICP). (B) Confocal images of ICP bread, starch (green), cell walls (blue), protein (red). Texture analyses (C): hardness (Newtons), gumminess (AU) chewiness (Newtons/s), springiness, cohesiveness and resilience (%), volume and bulk density. Palatability (D), visual analogue scale (VAS) scores (intensity %) of bread attributes. Values are means with 95% CI (n = 20). Bars with different lowercase letters (a, b, c) are significantly different to each other (p < 0.001, ANOVA with Tukey's *post-hoc* comparison).

All bread rolls had a similar appearance and size (Fig. 2A), regardless of the proportion of ICP used in the recipe. Colour analysis revealed that incorporation of ICP resulted in a darker, more yellow crumb (Fig. S.3 *–* ‘Results of colour analysis’). Imaging of breads incorporating ICP (Fig. 2B) demonstrated that cell structure was preserved following baking, and ICP was evenly dispersed throughout the crumb and crust in the 30% and 60% breads. There was a significant effect of ICP incorporation on bread crumb texture (Fig. 2C), with hardness, gumminess and chewiness (Fig. 2C.1) all increasing in a dose-dependent manner (*F*_2,11_ = 47,07, 87.12 and 62.35, *p* < 0.0001, respectively). Springiness, cohesiveness and resilience (Fig. 2C.2) decreased as the proportion of ICP increased (*F*_2,11_ = 11.59, 186.21, 87.83, *p* < 0.0001, respectively). Loaf volume showed a decrease with respect to ICP replacement level in the bread rolls, but the differences between breads were not statistically significant (Fig. 2C.3). However, bulk density (Fig. 2C.4) increased significantly as the proportion of ICP increased (0.25 ± 0 0.04, 0.357 ± 0.09, and 0.608 ± 0.101 g/L for the control, 30 and 60% bread types, respectively; *F*_2,6_ = 14.32, adj. *p* = 0.004).

Participant scores (n = 20) of bread attributes (Fig. 2D) provide the first indication of palatability. The enrichment of bread with ICP had no significant effects on aftertaste, texture, taste or enjoyment. Scores for moistness were significantly lower for 60% ICP bread rolls compared to control (*F*_2,57_ = 7.45, *p* = 0.001), and scores for ‘difficulty in consuming the given volume’ were significantly higher for the 60% ICP bread compared with the control (*F*_2,57_ = 5.09, *p* = 0.009). These scores could reflect the larger serving (mass) of 60% bread, compared to the 0% and 30% breads. These preliminary participant scores of bread quality attributes are encouraging, and further work will be needed to ascertain acceptable limits of wheat flour substitution with representative consumer groups.

### 3.3 Glycaemic effects and digestibility

Recent studies of common beans have shown that structural integrity, cell wall permeability and susceptibility to amylase depends on the processing conditions (Pallares Pallares et al., 2018), and it is well established that many resistant starch structures are liable to become more digestible upon hydrothermal processing (Roman & Martinez, 2019). A main objective in this study was therefore to establish if the novel ICP retained its slowly digestible starch and low glycaemic properties after secondary processing into a wheat bread product.

**Fig. 3:**
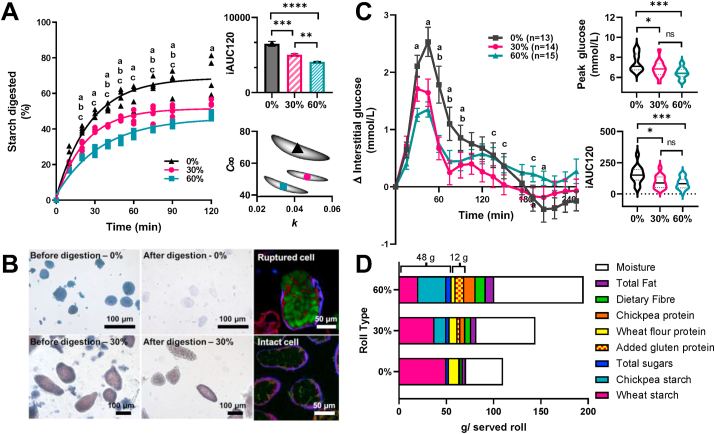
Glycaemic responses and bread digestibility. Starch amylolysis (A) curves, area under the curve (iAUC120) and parameter plots of *k* (rate constant) and C_∞_ (endpoint) for bread incorporating ▲0%, ●30% or ■ 60% ICP. Images (B), light micrographs show wheat starch in 0% bread and cells in 30% bread, before and after amylolysis. Confocal images show FITC-amylase (green) penetrating ruptured cell but not intact cells. Incremental glucose responses (C) to each bread as means ± SEM for number of participants (n) with insert violin plots showing peak glucose concentrations (NB, these are absolute values) and area under the first curve up to 120 min (not including area below baseline), iAUC120. Significant differences between bread types: ***a***, 0% vs 60%; ***b***, 0 vs 30% and ***c***, 30% vs 60%, are indicated at each time point by lowercase letters in panels A,C. Macronutrients measured in each bread roll type (D) are shown per roll (g/serving: ~115, 150 and 201 g for 0, 30 and 60%, respectively) as served to study participants. A consistent amount of total starch + sugar (~48 g) and total wheat protein (~12 g from added gluten and wheat flour) per served roll was achieved for all roll types; the amount of total protein, dietary fibre, fat and moisture per served roll varied between roll types.

*In vitro* starch amylolysis of bread rolls revealed for the first time, that starch encapsulated within the ICP retained its resistance following bread baking. ICP replacement of wheat flour significantly attenuated starch amylolysis profiles of the bread (*F*_2,9_ = 56.57, *p* < 0.001) and lowered iAUC_120_ values in a dose-dependent manner (Fig. 3A). Baseline-corrected first order amylolysis curves for the breads were specified by two parameters; the endpoint (*C*_*∞*_) and rate constant (*k*). The mean values and standard errors of these parameters obtained for the 0%, 30% and 60% bread types were *C*_*∞*_ = 68.3 ± 3.3, 51.4 ± 1.7 and 44.9 ± 1.2% and *k* = 0.040 ± 0.003, 0.045 ± 0.003 and 0.032 ± 0.002 min^−1^, respectively. The covariance of these parameters is shown in the parameter plots (Fig. 3A insert), which suggests that substitution of 30% wheat flour with ICP reduced the amount of starch digested (*C*_*∞*_), and that 60% substitution caused a reduction in rate of digestion as well as the extent. Furthermore, imaging (Fig. 3B) of bread roll samples and digesta showed that after 2 h *in vitro* digestion, chickpea cells remained intact, whereas only residual traces of wheat starch granule ghosts were observed. Confocal images confirmed that amylase (labelled with FITC) diffused into damaged cells, but not into intact cells. Together, these findings indicate that wheat, which provided ~75% and ~41% of the total starch in the 30 and 60% bread rolls, respectively, is the main source of starch amylolysis products in the *in vitro* assay, whereas the ICP starch enclosed within plant cells remains inaccessible to amylolysis despite further processing during bread making.

Glycaemic responses (measured by CGM) to 0, 30 or 60% (w/w) ICP bread rolls (each containing 48 g potentially available carbohydrate) were determined *in vivo* in healthy participants following a randomised cross over design in Study 2 (Fig. S.2– ‘CONSORT diagram’ and Table S.1 – ‘Participant Characteristics’). Using bread rolls formulated to contain the same amount of total starch per serving ensured that appropriate comparisons could be made between the *in vitro* digestion assays and the glycaemic study *in vivo.*

The breads containing ICP elicited considerably lower glycaemic responses compared with the control bread. There was a significant overall effect of ICP bread on glycaemia (Fig. 3C) (*F*_1.47,20.6_ = 5.03, *p* = 0.024), and a highly significant effect of time (*F*_3.14, 43.9_ = 27.33, *p* < 0.001) and a bread × time interaction (*F*_4.67, 57.0_ = 6.140, *p* < 0.001). The iAUC_120_ (Fig. 3C insert) were significantly lower after ingestion of ICP enriched bread compared with the control (*F*_1.67, 20.8_ = 13.27, *p* < 0.001), with 30% and 60% bread iAUC_120_ values ~40% lower than the control. These significant differences in relative glycaemic responses to ICP enriched bread rolls are explained by the lower bioaccessibility of starch from ICP seen *in vitro*.

Glycaemic reductions of a similar magnitude to our current study have been achieved by other groups through incorporation of whole or cracked grains/seeds into Scandinavian style breads (Björck, Granfeldt, Liljeberg, Tovar, & Asp, 1994; Liljeberg, Granfeldt, & Björck, 1992). However, the organoleptic properties of the test breads used in our study more closely resembled the type of white bread that is most common in the UK and US.

Interestingly, there were no significant differences between iAUC_120_ values for the 30% and 60% ICP breads. While an earlier study showed a complex dose-dependent relationship for ICP incorporation on starch digestibility of biscuits (Delamare et al., 2020), the processing conditions during bread making provide a higher moisture environment than the biscuit baking process, favouring wheat starch swelling and gelatinisation (Wootton & Chaudry, 1980), and loss in starch resistance (Fardet et al., 2006; Roman & Martinez, 2019). Further studies should explore the dose-response relationship in bread at intermediate doses.

There was a significant effect of bread type on maximum, ‘peak’, glucose concentrations (Fig. 3C) (*F*_1.82, 22.8_ = 12.51, *p* < 0.001), with lower peaks after ingestion of ICP enriched breads (mean = 6.86 ± 0.81 mmol/L, n = 14, for 30% bread and 6.44 ± 0.56 mmol/L, n = 15, for 60% bread) compared to control (7.49 ± 0.87 mmol/L, n = 13). Mean glucose concentrations peaked at ~45 min irrespective of bread type (*F*_1.47, 18.4_ = 0.119, *p* = 0.827). Additionally, a second glucose peak was observed between 90 and 120 min after consumption of the 30 and 60% breads, and based on the *in vitro* starch amylolysis kinetics, we can speculate that the first glucose peak may reflect the highly digestible wheat starch, whereas the second peak could reflect the gradual and sustained availability of glucose from digestion of starch in the ICP. The shape of the postprandial curve is more relevant for interpretation of postprandial pathophysiology and appetite than the iAUC (Berry et al., 2020; Chung et al., 2017; de Andrade Mesquita, Pavan Antoniolli, Cittolin-Santos, & Gerchman, 2018). The flattening of the postprandial glucose response represents a potential strategy for improving glucose homeostasis in people at risk to or with type 2 diabetes and should be investigated. In our study the glucose curve beyond 180 min for the control bread showed negative values (i.e. it dips below the baseline), while there was a tendency for ICP enriched breads to remain closer to baseline. Emerging evidence suggests that a smaller ‘dip’ below baseline (seen for the ICP breads compared with control) is linked to reduced hunger and calorie intake (Berry et al., 2020), which is of relevance to the area of obesity prevention.

It is noteworthy that the test breads contained similar amounts of total starch and wheat protein per serving, but differed in total protein, fat and dietary fibre content (Fig. 3D and Table 2). Bioavailable protein may stimulate gut incretins and thereby influence postprandial glycaemia (Pais, Gribble, & Reimann, 2016); however, micrographs of *in vitro* digesta show that some protein remains encapsulated together with the starch, hence, the intracellular protein from ICP is not readily bioavailable. Our amylolysis assay was designed for investigation of starch digestion kinetics and did not include proteases, but previous *in vitro* studies suggest that leguminous plant cell walls may also hinder the ingress of proteases (Bhattarai et al., 2017; Rovalino-Córdova et al., 2019; Würsch et al., 1986). *In vivo,* intact legume cells have been identified at the terminal ileum (Noah et al., 1998) and in aspirates recovered from the upper gastrointestinal tract of healthy humans (Petropoulou et al., 2020). It therefore seems plausible that the protein in ICP remains inaccessible during early stages of digestion. These observations highlight the importance of considering not just nutrient composition of the test meals, but their spatial location and digestion behaviour when interpreting results. Indeed, there is now convincing evidence that cell wall encapsulation of nutrients is a key mechanism by which dietary fibre influences postprandial metabolism (Ellis et al., 2004; Grundy et al., 2016; Holland, Ryden, Edwards, & Grundy, 2020).

From a consumer perspective, it is more relevant to compare nutrient composition per 100 g matched servings (Table S2). When matched by weight, ICP enriched breads contain less total starch and more dietary fibre and chickpea protein than the control wheat bread. As the ICP is gluten-free, more gluten was added to the recipe when replacing higher proportions of wheat flour. However, in the final baked product, the total wheat protein content (mainly gluten) per 100 g was 37% lower in the 60% bread roll than in the control bread. Gastrointestinal symptoms associated with non-coealiac gluten sensitivity are relatively common (~13% of UK adults), so the potential new use of ICP for low-gluten applications merits further investigation (Lebwohl, Ludvigsson, & Green, 2015). On the other hand, chickpeas do contain oligosaccharides and other fermentable carbohydrates which seem to contribute to flatulence in some individuals (Thompson, 2019; Winham & Hutchins, 2011). No gastrointestinal symptoms were reported by participants consuming chickpea products in the present study, but the gastrointestinal effects of chronic intake should be explored.

ICP-enriched breads contain less starch per 100 g and therefore provide a lower glycaemic load than the wheat control bread. In the 60% bread roll, approximately 60% of the total starch is in the form of ICP. Analytically, the 60% bread roll contained 1.55 g of RS per 100 g; however, based on the digestibility profile of the ICP, up to ~12 g/100 g could be Type 1 resistant starch. Further work is needed to clarify how such fractions are best captured by new and emerging definitions and methods of analyses for nutritionally important carbohydrates.

Reducing the glycaemic potency of white bread as a starch staple food product that is eaten by many (Pot, Prynne, Almoosawi, Kuh, & Stephen, 2015), is an integral part of public health strategies (World Health Organization, 1998) and would facilitate dietary management of people with or at risk of type 2 diabetes. Overall, this work has wide implications for both fundamental and applied research including addressing global strategical challenges, e.g., in diabetes care, crop improvement and food security. Incorporation of this novel cellular flour into bread and other staple foods provides a new opportunity to develop the next generation of low glycaemic food products, and would also provide a new means of increasing dietary fibre, resistant starch and legume protein intakes to support public health measures (Bazzano et al., 2001; Venn & Mann, 2004).

## 4 Conclusions

This study utilised a combination of *in vitro* and *in vivo* techniques to demonstrate the glycaemic potential and underpinning mechanisms of a novel cellular ‘flour’ incorporated into a staple food. We have confirmed the resilience of the plant cells in this powder to secondary processing and demonstrated that replacement of wheat flour with chickpea cell powder in a conventional wheat bread recipe produces a more favourable glycaemic response to white bread. This study also reveals that plant cell integrity is the critical factor limiting the bioaccessibility of starch from pre-processed powders. Thus, the proprietary process used to obtain cellular flours provides a new route to preserving the beneficial structure of dietary fibre (i.e. intact cell walls with limited permeability to amylase) that underpins the low glycaemic effects of whole pulses into bread and other highly processed food products. The results of the acute metabolic study provide strong justification for undertaking further chronic intervention trials of bread and other products enriched with cellular legume powders on glycaemic control in pre-diabetic and diabetic cohorts.

## CRediT authorship contribution statement

**Balazs H. Bajka:** Conceptualization, Formal analysis, Data curation, Writing - original draft, Writing - review & editing, Supervision, Project administration. **Ana M. Pinto:** Formal analysis, Investigation, Data curation, Writing - original draft, Writing - review & editing. **Jennifer Ahn-Jarvis:** Formal analysis, Investigation, Resources, Data curation, Writing - original draft, Writing - review & editing. **Peter Ryden:** Formal analysis, Investigation, Resources, Data curation, Writing - original draft, Writing - review & editing. **Natalia Perez-Moral:** Investigation, Data curation, Writing - original draft, Writing - review & editing. **Alice van der Schoot:** Investigation, Data curation. **Costanza Stocchi:** Investigation, Data curation. **Catherine Bland:** Investigation, Data curation. **Sarah E. Berry:** Conceptualization, Writing - review & editing, Supervision, Funding acquisition. **Peter R. Ellis:** Conceptualization, Writing - review & editing, Project administration, Supervision, Funding acquisition. **Cathrina H. Edwards:** Conceptualization, Writing - original draft, Writing - review & editing, Formal analysis, Visualization, Project administration, Supervision, Funding acquisition.

## Declaration of competing interest

There are no immediate conflicts of interest. The methods of preparation of chickpea cell powder (as PulseON®) is covered by a published international patent application but is not commercialised. The patent is authored by CE and PE. SB receives consultancy and shares from ZOE Global Ltd. The other authors [BB, AP, PR, JAJ, NPM, AS, CS, CB] have no conflicts of interest to declare.
