# Supplementary material for: The impact of replacing wheat flour with cellular legume powder on starch bioaccessibility, glycaemic response and bread roll quality: A double-blind randomised controlled trial in healthy participants
Source: Food Hydrocoll. 2021 May;114:106565. doi: 10.1016/j.foodhyd.2020.106565 (PMC7859705; doi:10.1016/j.foodhyd.2020.106565)
Supplement: Supplementary file 1 [file mmc1.pdf]

Supplementary Material

Table S1: Characteristics of the participants who enrolled in each study[1]

	STUDY 1 (n = 20)	STUDY 2 (n = 21)
Age (years)	26.0 ± 4.08	27.9 ± 4.91
Gender	8 males;<br>12 females	10 males;<br>10 females
Ethnicity	White 10<br>Asian 7<br>Mixed 1<br>Black 2	White 14<br>Asian 4<br>Mixed 2
Body mass index (kg/m^2)	23.8 ± 4.67	24.7 ± 3.1
Systolic /diastolic blood pressure (mmHg)	106.3 ± 10.67<br>/ 70.8 ± 7.43	111.1 ± 12.77<br>/75.3 ± 7.62
Fasted plasma glucose (mmol/L)	4.79 ± 0.43	4.78 ± 0.40
Serum triacylglycerols (mmol/L)	0.74 ± 0.19	0.89 ± 0.58
Serum total cholesterol (mmol/L)	4.63 ± 0.84	4.62 ± 1.04

[1]Values are means ± SD. Ethnicity was self-reported.

Figure S1: Study 1 CONSORT diagram

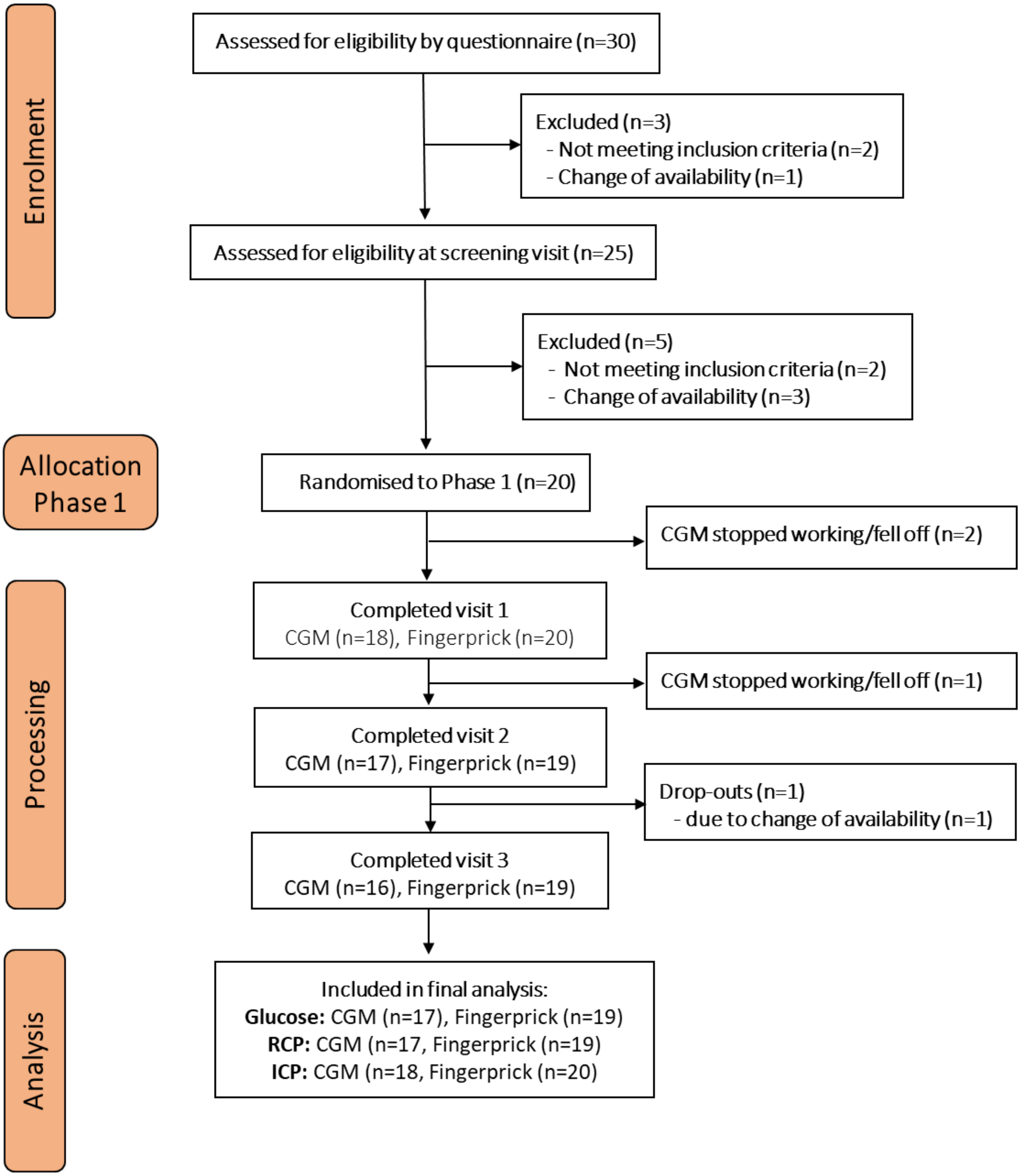

Figure S2: Study 2 CONSORT diagram

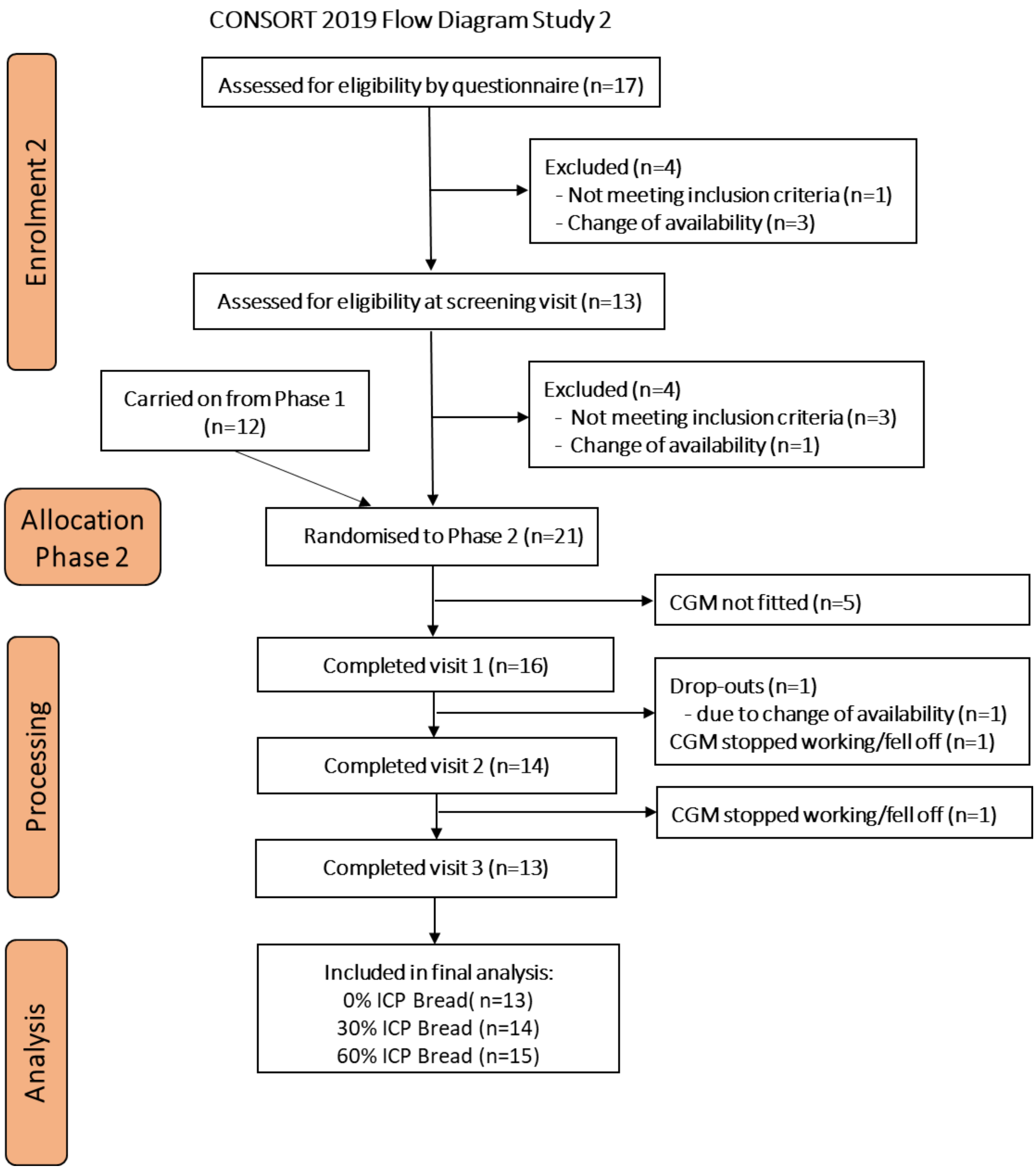

Table S2: Nutrient composition (g/100g as served) of bread rolls made with 0, 30 and 60% intact cell powders (ICP)

	0% ICP	30% ICP	60% ICP
Moisture (g) [1]	34.0	41.5	47.0
Energy (kJ) [1]	1132.0	1001.0	906.1
Protein (g)	11.2	11.8	13.5
from wheat [3]	94%	65%	49%
from chickpea [3]	0%	29%	44%
Total Fat (g) [1]	2.9	3.7	4.4
Saturated fatty acids (g) [1]	*0.87*	*0.96*	*1.05*
Monounsaturated fatty acids (g) [1]	*1.04*	*1.27*	*1.49*
Polyunsaturated fatty acids (g) [1]	*0.86*	*1.31*	*1.67*
Total Starch (g) [2]	39.4	29.9	21.1
from wheat [3]	100%	75%	41%
from chickpea [3]	0%	25%	59%
Digestible starch [2]	99%	96%	93%
Resistant starch [2]	1%	4%	7%
Total Sugars (g)	2.4	2.3	2.8
Potentially available carbohydrates (g) [4]	41.8	32.2	23.9
Dietary Fibre (g) [1]	2.3	4.1	5.3
Sodium (mg)	364.0	362.0	373.6

[1]Proximate determinations by ALS Laboratories, [2]Direct measurements obtained using Megazyme total and resistant starch kits. [3]Estimated proportion of protein or starch originating from wheat (wheat flour, gluten) and chickpea (ICP) calculated form ingredient composition and amounts used in each recipe, [4] 'Potentially available carbohydrates' is the sum of total starch and sugars. Data is shown for 0, 30 and 60% bread roll types, where the % refers to proportion of wheat flour replaced with ICP (w/w).

Figure S3: Results of colour analysis of bread rolls made with 0, 30 or 60% intact cell powders

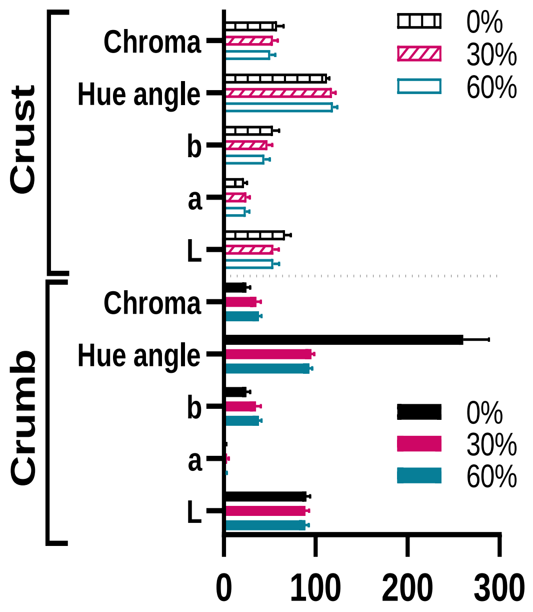

Supplementary Data 1:

Postprandial interstitial glucose responses to test drinks

DRINK	**GLUCOSE DRINK**			**RUPTURED CELL POWDER**			**INTACT CELL POWDER**		
Time (min)	Mean	SD	n	Mean	SD	n	Mean	SD	n
15	1.05	0.55	17	0.68	0.46	17	0.38	0.33	18
30	2.80	0.93	17	1.74	0.91	17	1.19	0.55	18
45	3.23	1.46	17	1.81	1.18	17	1.57	0.70	18
60	2.43	1.61	17	1.05	0.95	17	1.26	0.64	18
75	1.60	1.47	17	0.48	0.53	17	0.81	0.52	18
90	1.18	1.28	17	0.19	0.44	17	0.44	0.51	18
105	0.91	1.16	17	0.09	0.57	17	0.13	0.53	17
120	0.56	1.04	16	0.07	0.53	16	0.05	0.42	16

Supplementary Data 2:

Postprandial capillary glucose responses to test drinks

DRINK	**GLUCOSE DRINK**			**RUPTURED CELL POWDER**			**INTACT CELL POWDER**		
Time (min)	Mean	SD	n	Mean	SD	n	Mean	SD	n
10	0.84	0.55	19	0.19	0.43	18	0.17	0.35	18
20	2.37	1.14	19	1.18	0.64	19	0.68	0.64	20
30	2.84	1.02	19	1.73	1.14	19	1.31	0.81	20
45	2.45	1.42	19	1.36	1.10	19	1.24	0.96	19
60	2.06	1.38	19	0.69	0.75	18	1.04	0.96	20
90	1.16	1.25	19	0.26	0.72	19	0.50	0.72	20
120	0.67	1.08	19	0.00	0.72	19	0.09	0.61	20

Supplementary Data 3:

Postprandial interstitial glucose responses to bread rolls

BREAD TYPE	**0% BREAD ROLL**			**30% BREAD ROLL**			**60% BREAD ROLL**		
Time (min)	Mean	SD	n	Mean	SD	n	Mean	SD	n
0	0.00	0.00	13	0.00	0.00	14	0.00	0.00	15
15	0.65	0.48	13	0.72	0.61	14	0.40	0.38	15
30	2.11	0.69	13	1.71	0.82	14	1.26	0.44	15
45	2.53	0.92	13	1.64	0.89	14	1.35	0.52	15
60	1.78	1.16	13	0.67	0.74	14	0.75	0.57	15
75	1.10	1.05	13	0.25	0.80	14	0.44	0.75	15
90	0.85	0.80	13	0.38	0.83	14	0.45	0.65	15
105	0.75	0.69	13	0.40	0.69	14	0.52	0.50	15
120	0.68	0.71	13	0.26	0.67	14	0.58	0.67	15
135	0.55	0.78	13	0.18	0.71	14	0.51	0.68	15
150	0.36	0.79	13	0.04	0.64	14	0.38	0.54	15
175	0.06	0.71	13	-0.06	0.59	14	0.24	0.58	15
190	-0.20	0.82	13	-0.16	0.56	14	0.22	0.53	15
205	-0.39	0.81	13	-0.18	0.65	13	0.15	0.49	15
220	-0.38	0.66	13	-0.12	0.77	13	0.06	0.48	14
235	-0.28	0.60	13	-0.08	0.77	13	0.13	0.49	14
250	-0.25	0.62	13	-0.07	0.73	13	0.27	0.81	14
